# Effect of peripheral iridectomy on VEGF-A and TGF-β levels in rabbit aqueous humour

**DOI:** 10.1186/s12886-022-02249-6

**Published:** 2022-01-14

**Authors:** Annabel C. Y. Chew, Li-Fong Seet, Stephanie W. L. Chu, Nyein C. Lwin, Tina T. Wong

**Affiliations:** 1 Singapore National Eye Centre, 11 Third Hospital Avenue, Singapore, 168751 Singapore; 2 Singapore Eye Research Institute, Singapore, Singapore; 3 Department of Ophthalmology, Yong Loo Lin School of Medicine, National University of Singapore, Singapore, Singapore; 4 Duke-NUS Medical School Singapore, Singapore, Singapore

**Keywords:** Peripheral iridectomy, Aqueous TGF-β1, Aqueous TGF-β3

## Abstract

**Background:**

Peripheral iridectomy (PI), routinely performed during glaucoma filtration surgery, may contribute to scarring. This study aims to determine whether PI alters the concentrations of VEGF-A and TGF-β isoforms in the rabbit aqueous humour.

**Methods:**

Anterior chamber paracentesis (ACP) was performed in both eyes of six New Zealand white rabbits, with additional surgical PI performed in the right eyes. Eyes were examined on postoperative days (PODs) 1, 7, 30 and 60 by means of the tonopen, slit-lamp biomicroscopy, and bead-based cytokine assays for TGF-β and VEGF-A concentrations in the aqueous humor.

**Results:**

ACP caused a significant reduction in intraocular pressure (IOP) from mean preoperative 11.47 ± 1.01 mmHg to 5.67 ± 1.63 mmHg on POD 1 while PI did not cause further IOP reduction. Limbal conjunctival vasculature appeared slightly increased on POD 1 in both ACP and PI eyes with PI also causing mild bleeding from damaged iris vessels. Two PI eyes developed fibrinous anterior chamber reaction and/ or peripheral anterior synechiae. Aqueous VEGF-A levels were not significantly different between eyes treated with ACP and PI. Aqueous TGF-β concentrations distributed in the ratio of 4:800:1 for TGF-β1:TGF-β2:TGF-β3 respectively. While aqueous TGF-β2 was not significantly induced by either procedure at any time point, TGF-β1 and TGF-β3 were significantly induced above baseline levels by PI on POD 1.

**Conclusion:**

PI increases the risk of inflammation. The combined induction of aqueous TGF-β1 and TGF-β3 by PI in glaucoma surgery may impact surgery success in glaucoma subtypes sensitive to these isoforms.

## Synopsis

Peripheral iridectomy increases the incidents of inflammatory responses as well as induces aqueous TGF-β1 and TGF-β3, but not TGF-β2 or VEGF-A.

## Introduction

Subconjunctival fibrosis is the most common cause of postoperative failure in glaucoma filtration surgery. Indeed, increased bleb vascularity is associated with poor surgical prognosis [1]. As a component of wound repair due to tissue damage, angiogenesis occurs to recover tissue homeostasis [2]. However, excessive angiogenesis may contribute to the development of fibrosis [3]. The angiogenic process is complex and a number of growth-promoting factors are known to regulate its induction. The most potent proangiogenic molecules include members of the Vascular Endothelial Growth Factor (VEGF) family. The prototype member, VEGF-A, is considered the master regulator of angiogenesis [4]. VEGF-A has the capacity to stimulate endothelial cell proliferation, migration and coalescence to form larger vessels [5]. Importantly, VEGF-A was demonstrated to directly promote the growth activity of conjunctival fibroblasts, and further implicated in modulating scarring in a rabbit model of glaucoma surgery [6]. In corroboration, higher preoperative VEGF levels in Tenon’s tissue are associated with a worse outcome following glaucoma surgery [7]. Current experimental and clinical evidence favors a role for VEGF-A in modulating fibrosis in glaucoma surgery [8]. As VEGF-A levels are elevated in the aqueous humor of glaucomatous eyes [6, 7], and may be further upregulated following glaucoma surgery [6, 9], the surgical success may be dependent on limiting VEGF-A induction.

The TGF-β family, consisting of three isoforms (TGF-β1, −β2, and –β3), has been recognized as a key mediator of the fibrotic response in wound healing [10]. All three isoforms have the capacity to stimulate Tenon’s capsule fibroblast-mediated collagen contraction, proliferation, and migration [11]. In a mouse model of conjunctival scarring, all three isoforms caused an exaggerated scarring [12]. Conversely, TGF-β2 neutralization in conjunctival fibroblasts and in rabbit models of glaucoma surgery reduced pro-fibrotic cellular activities and subconjunctival scarring with improved surgical outcome, supporting the involvement of TGF-β2 as a mediator of fibrosis in glaucoma surgery [13]. Clinically, glaucoma patients were reported to harbor higher conjunctival TGF-β levels, with greater reduction after augmented trabeculectomy being associated with surgical success [14]. Elevated levels of TGF-β2 were detected in the aqueous humor of glaucomatous eyes compared with normal eyes [15], with non-elevated TGF-β2 levels being associated with favorable bleb development [16]. We have previously reported that experimental surgery in a mouse model of conjunctival scarring increased the expression of both *Tgfb1* and *Tgfb2* mRNAs [17], indicating that the surgery itself, involving the creation of a fistula for aqueous humor drainage into the subconjunctiva [18], may stimulate TGF-β production. Moreover, exogenous VEGF-A introduced into the anterior chamber (AC) was demonstrated to induce subconjunctival TGF-β1 in a rabbit model of glaucoma surgery [9]. These findings suggest the potential existence of multiple sources of stimulation for TGF-β induction in glaucoma surgery, including the procedure itself as well as elevated VEGF-A levels associated with glaucoma. Hence, a better understanding of the surgical procedure as a possible provocateur for further rise in already higher levels of VEGF-A and TGF-β associated with glaucoma, will be helpful towards improving surgical success.

In glaucoma filtration surgery, peripheral iridectomy (PI) is routinely performed to prevent iris occlusion of the sclerostomy. It may be associated with complications such as bleeding, zonular or lens disruption, increased inflammation, iridodialysis and posterior synechia [19]. The role of PI in contributing to postoperative complications and fibrosis warrants investigation. The primary goal of this study was to evaluate whether PI alters the levels of aqueous humor VEGF-A and TGF-β isoforms. The secondary goal involved the assessment of other effects of PI including the development of inflammatory reactions.

## Materials and methods

### Rabbit model of PI

The animal experiments were approved by the Institutional Animal Care and Use committee (IACUC) and treated in accordance with the Association for Research in Vision and Ophthalmology (ARVO) Statement on the Use of Animals in Ophthalmic and Vision Research. Six female New Zealand White rabbits aged 3 to 4 months old and weighing between 2 and 2.5 kg were used for this study. We have chosen to use 6 rabbits in this study to obtain high quality and reproducible data for our experimental aims. Experimental studies involving laboratory animals generally consist 3 to 5 independent samples due to use of genetically homogeneous, in-bred animals, living in highly controlled environments. A sample size of 5 is suggested to be the minimum number required for experimental studies in animals [20] . The rabbits were procured from InVivos Pte Ltd., a supplier of research animals in Singapore. The rabbits used were healthy, not genetically modified and had no previous procedures performed.

Surgery was performed by a single surgeon in the animal laboratory in the Singapore Eye Research Institute. The rabbits were anesthetized with an intramuscular injection of ketamine (40 mg/kg)/ xylazine (4 mg/kg), and topical xylocaine was instilled into the eyes. The periocular area was cleaned with 10% povidone-iodine and a wire lid speculum placed to separate the eyelids. 5% povidone-iodine was applied onto the ocular surface for a few minutes before the surgery. In the left eye, anterior chamber paracentesis (ACP) was performed by making a 1.0 mm limbal incision in the superior quadrant using a 15-degree blade. In the right eye, ACP was performed, followed by removal of a small section of the iris using Vannas scissors. In both eyes, the anterior chambers were reformed using balanced salt solution (BSS) and the wounds were hydrated. Postoperative care included daily treatment with topical maxitrol 4 times a day (Alcon Laboratories, Fort Worth, Texas, USA) in both eyes for a week after the surgery. Rabbits were followed up on post-operative day (POD) 1, 7, 30 and 60. Rabbits were sacrificed on post-operative month 3 via intravenous injections of sodium pentobarbitol (100 mg/kg).

### Intraocular pressure (IOP) measurements

Intraocular pressure (IOP) were measured using a tonopen (Tono-Pen AVIA Reichert) pre-operatively and at each post-operative time point. Triplicate measurements of each eye at each time point were recorded.

### Slit lamp biomicroscopy

Rabbits were anaesthetized before imaging by slit lamp biomicroscopy pre-operatively, and at each post-operative time point. Slit lamp imaging was performed using Righton LED slit lamp MW50D (Right Mfg Co Ltd., Japan).

### Aqueous cytokine analysis

AC taps were performed pre-operatively and at each post-operative time point using aseptic technique with 30G needle to obtain 100 μl aqueous humor from each eye. Aqueous humor samples were stored in − 80 °C before analyses. Cytokine assay was performed using the MILLIPLEX MAP Magnetic Bead Kit (Merck Millipore, Billerica, MA) according to instructions by the manufacturers. Cytokine levels were measured using the Bio-Plex 200 system (Bio-Rad Laboratories, Hercules, CA) and values were normalized to total protein content of each sample.

### Statistical analysis

All the statistical analyses were performed using the SPSS version 19 software (IBM SPSS Statistics for Windows, Version 19.0. Armonk, NY: IBM Corp. Released 2010). All data are expressed as mean ± standard deviation (SD). The significance of differences between the conditions was determined by one-way ANOVA using SPSS statistics. Bonferroni post-hoc adjustment was applied to determine which conditions were significantly different from each other. Statistical significance was defined as *p* < 0.05.

The surgeon was aware of the group allocation. The research assistants who measured the IOP and took the slit lamp photos, and the scientist who performed the aqueous cytokine analysis were not aware of the group allocation. The study was reported in accordance with ARRIVE guidelines.

## Results

### Effects of PI on IOP

**Fig. 1:**
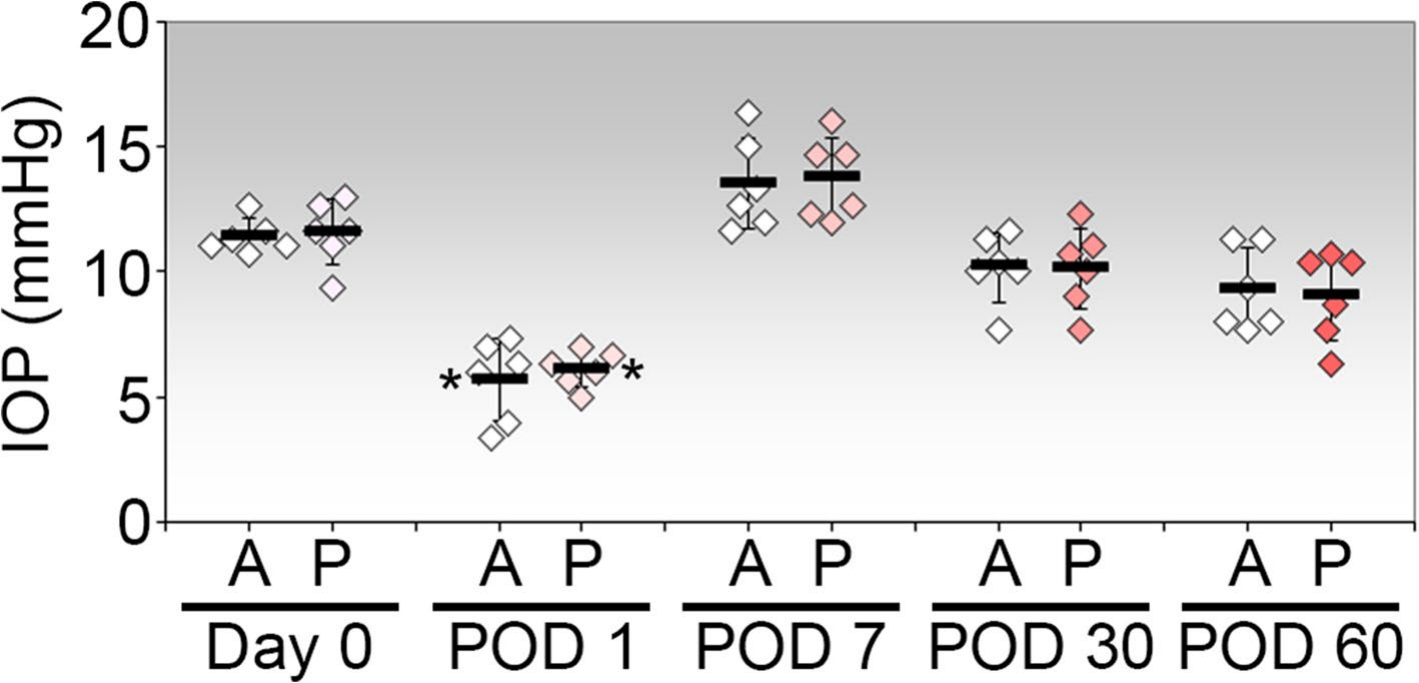
Intraocular pressures in rabbit eyes treated with ACP (*n* = 6) or ACP in conjunction with PI (*n* = 6). Tonopen measurements were recorded at the indicated times. Each point represents the IOP of one eye and is the average of triplicate measurements. The mean IOP ± SD for each treatment and time point is shown. IOPs measured on day 0 prior to ACP are considered preoperative baseline values for statistical comparison. *, *P* < 0.05 (Bonferroni-adjusted) relative to the respective baseline value on day 0. A, ACP only in left eye; P, ACP with PI in contralateral eye

The preoperative IOPs were similar in both eyes of each rabbit, with the mean preoperative IOP of all 12 eyes of 6 rabbits being 11.47 ± 1.01 mmHg (Fig. 1). ACP alone caused a significant reduction in IOP by about 50% to 5.67 ± 1.63 mmHg on POD 1 (*p* = 0.00000080; 95% CI: 2.77–8.68; Fig. 1). The additional PI procedure resulted in IOP decrease to 6.11 ± 0.72 mmHg on POD 1(*p* = 0.0000026; 95% CI: 2.49–8.40; Fig. 1), which was not significantly different from ACP alone. IOPs in both eyes returned to levels that were not significantly different from the baseline levels when measured from POD 7 onwards. Hence, ACP alone caused significant acute but temporary IOP reduction while the additional procedure with PI did not cause further IOP reduction in normal rabbit eyes.

### Effects of PI on Limbal Conjunctival vascularization

**Fig. 2:**
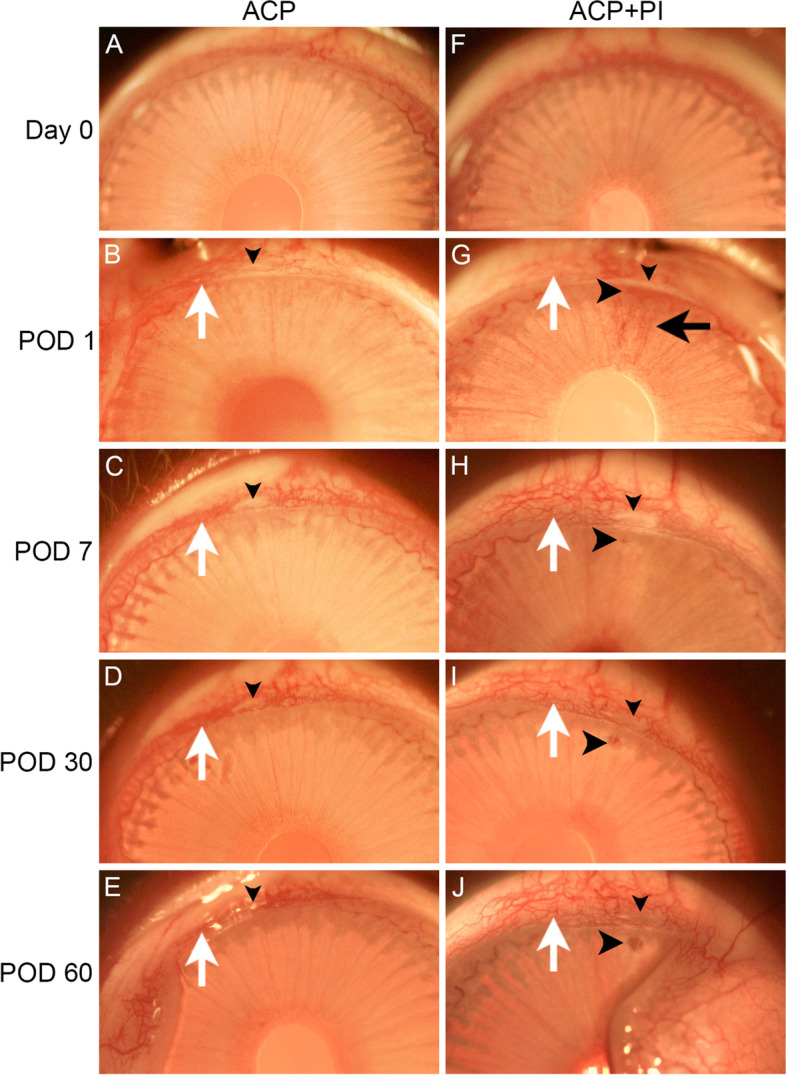
Slit lamp biomicroscopy of rabbit eyes treated with ACP or ACP in conjunction with PI. (**A**-**E**) Anterior segment images of the left eye of a rabbit treated with ACP only at the indicated time points. (F-J) Anterior segment images of the contralateral right eye of the same rabbit treated with ACP and PI at the indicated time points. Small black downward arrowhead indicates the ACP wound site. Large black rightward arrowhead indicates the PI wound site. White upward arrow indicates exemplary increase in limbal conjunctival vasculature. Black leftward arrow indicates mild bleeding from the injured iris observable only on POD 1

Examination by slit lamp biomicroscopy revealed that ACP alone generally spared vessels of the corneal arcade which appeared largely undisturbed (Fig. 2A-E). However, the limbal conjunctival vasculature appeared slightly increased on POD 1 (Fig. 2B), with no further increase when observed from POD 7 onwards (Fig. 2C-E). Similarly, slightly increased vessels in the limbal conjunctival plexus without in-growth of blood vessels from the pericorneal plexus into avascular corneal tissue were observed in the contralateral eye subjected to additional PI on POD 1 (Fig. 2F, G). Notably, any increase in vessels in the limbal conjunctival plexus were non-progressing and stable when examined for up to 2 months post-PI (Fig. 2H-J). Mild bleeding from damaged iris vessels may be observed after PI, but this was evident mainly on POD 1 (Fig. 2G) which then resolved spontaneously without the development of gross hyphema. The PI wound remained observable in follow-ups up to 2 months (Fig. 2J). Overall, PI did not appear to induce visibly greater increase in limbal vessels compared to ACP alone.

### Effects of PI on inflammatory responses

**Fig. 3:**
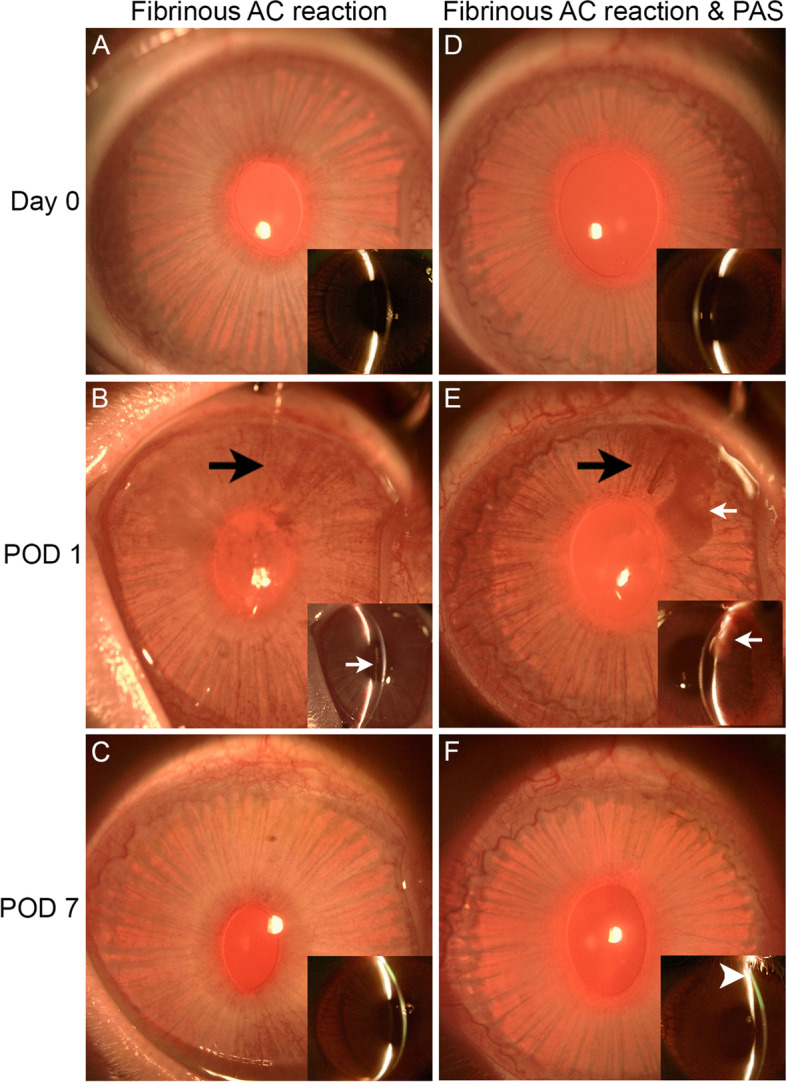
Slit lamp biomicroscopy of two rabbit eyes demonstrating PI-associated inflammatory responses. (**A**-**C**) Anterior segment images of one rabbit eye which displayed fibrinous anterior chamber reaction on POD 1 (inset, white arrow), with apparent resolution by POD 7. (**D**-**F**) Anterior segment images of a second rabbit eye which displayed fibrinous anterior chamber reaction on POD 1 (main image and inset, white arrow), and the development of peripheral anterior synechiae (PAS) on POD 7 (inset, white arrowhead) when fibrin coagulum was no longer apparent. Mild bleeding from the operated iris in the vicinity of the PI site was detected in both eyes on POD 1 (black arrow) but which appeared to have resolved by POD 7. Insets are narrow slit beam images of the same eyes focused on the pathological changes at the indicated time points

PI was associated with other responses, including fibrinous anterior chamber reaction and peripheral anterior synechiae (PAS). While no fibrinous anterior chamber reaction was detected in the ACP only eyes, two eyes with PI developed fibrinous anterior chamber reactions on POD 1 (Fig. 3A-C, D-F), which resolved by POD 7 (Fig. 3C, F). In one of the eyes, a flocculent fibrovascular fibrinous membrane in the AC that appeared to distort the iris was observed on POD 1 (Fig. 3E) prior to the development of PAS immediately inferior to the PI site on POD 7 (Fig. 3F). No other eyes developed PAS and no other complications were detected. The detection of fibrinous anterior chamber reactions and PAS only in the PI eyes suggest that there was more early postoperative intraocular inflammation in these eyes compared to eyes which had undergone ACP alone.

### Effects of PI on aqueous VEGF-A and TGF-β

**Fig. 4:**
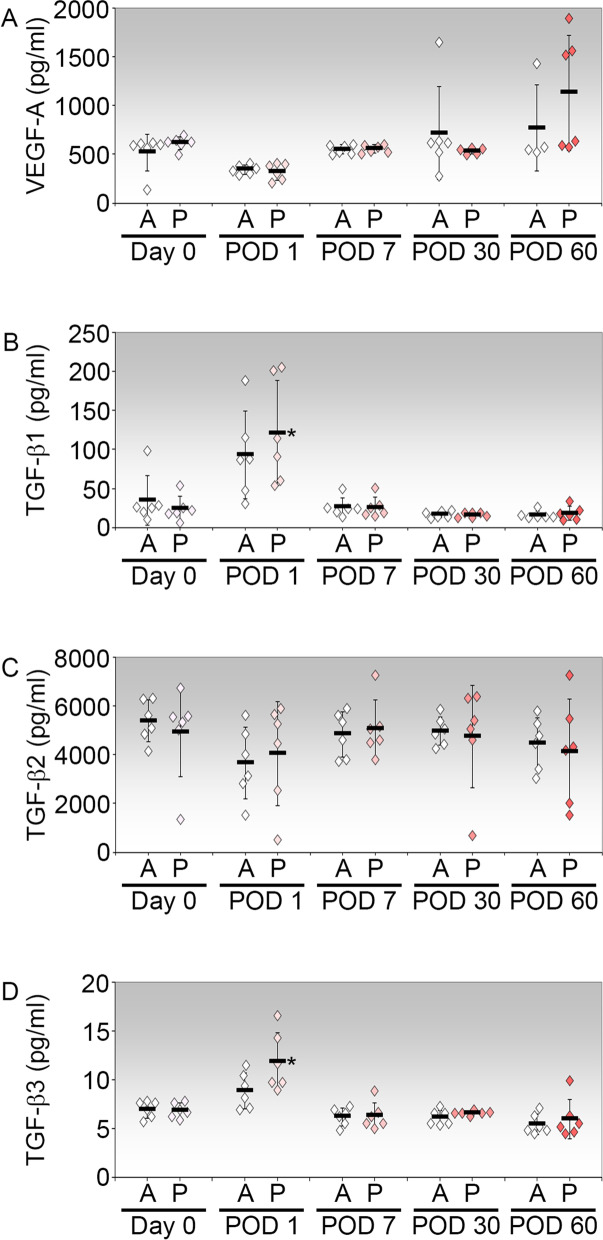
Concentrations of VEGF-A and TGF-β isoforms in the rabbit aqueous humour. Each point represents the concentration in one eye. Where there were less than 6 data points, the absent values were beyond the threshold of detection assigned by the assay. The mean concentration ± SD for each treatment and time point is shown. Cytokine concentrations in aqueous humor collected on day 0 via ACP are considered preoperative baseline values for statistical comparison. *, P < 0.05 (Bonferroni-adjusted) relative to the respective baseline value on day 0. A, ACP only in left eye (*n* = 6); P, ACP with PI in contralateral eye (*n* = 6)

The levels of VEGF-A and TGF-β isoforms in the aqueous humor of individual eyes were measured via Luminex-based magnetic bead assays. VEGF-A levels were not significantly different between the procedures, or when compared across time points up till POD 60 (Fig. 4A).

Aqueous TGF-β isoforms, on the other hand, demonstrated distinctly different profiles in their responses to the procedures. First, all three TGF-β isoforms were present in the preoperative aqueous humor, with baseline TGF-β2 being present at the greatest amount, at approximately mean 200- and 800-fold higher than TGF-β1 and TGF-β3 respectively (Fig. 4B-D). Although ACP alone appeared to cause a rise in TGF-β1 concentrations on POD 1, the mean increase was not significant compared to the baseline level. In comparison, the increase in TGF-β1 concentration due to PI was significantly higher than baseline by mean 5.04-fold on POD 1 (*p* = 0.000066; 95% CI: 35.59–158.50; Fig. 4B). Apart from that, TGF-β1 concentrations in the aqueous humor of PI eyes were not significantly different from that in ACP-treated contralateral eyes at any time points, and appeared relatively stable at levels near to baseline in subsequent time points. In contrast, there was no apparent induction of TGF-β2 by either procedure at any time point measured. Although TGF-β2 levels appeared to show relatively large deviations within each procedure at each time point, the mean TGF-β2 levels were largely similar between procedures and across time points (Fig. 4C). Similar to TGF-β1, while ACP appeared to cause a small rise in TGF-β3 concentrations on POD 1, this elevation was insignificant compared to the significant mean 1.73-fold spike from baseline measured in the contralateral eyes with PI (0.000023; 95% CI: 1.99–7.98; Fig. 4D). When analysed from POD 7 onwards, TGF-β3 levels had returned to near preoperative levels and were relatively stable up to POD 60. Taken together, PI caused the acute significant induction of aqueous TGF-β1 and TGF-β3 in the immediate aftermath of the procedure while VEGF-A and TGF-β2 did not appear to be modulated by either ACP or PI.

## Discussion

We have demonstrated in this study that PI elevates the inflammatory response compared to ACP alone, which manifested as fibrinous anterior chamber reaction and peripheral anterior synechiae (PAS). The selective induction of aqueous humor TGF-β1 and TGF-β3 by PI suggests this procedure may molecularly impact the success of glaucoma surgery by inducing postoperative fibrosis.

Fibrin reaction may result from increased vascular permeability caused by surgical procedures. It typically occurs in the early postoperative period and responds well to steroid therapy, resolving within 1 day to 3 weeks, supporting inflammation as an underlying cause. Hence, PI may increase intraocular inflammation, resulting in fibrinous AC reaction in the affected rabbit eyes.

Similarly, peripheral anterior synechiae (PAS) is commonly associated with AC inflammation. PAS has been observed in a variety of ocular conditions including uveitis, neovascular glaucoma, iridocorneal endothelial syndrome, as well as following trauma and surgeries. Patients typically display symptoms associated with underlying active or prior inflammatory processes. The deposition of inflammatory cells, fibrin and proteins, which is thought to stimulate the formation of adhesions between structures, is central to PAS pathophysiology. Overall, the increased incidents of PAS associated with PI in rabbits corroborate observations of increased inflammation in patients who have had this procedure [19].

We did not find a significant difference between aqueous VEGF-A levels in eyes with PI compared to ACP alone. Even though PI did not increase aqueous VEGF-A, it is still possible for PI to increase VEGF-A in other sites. Local VEGF-A elevation in the iris may contribute to iridal inflammation that potentially promotes fibrinous anterior chamber reaction and PAS. Nonetheless, we expect that the lack of elevation of aqueous VEGF-A will be beneficial in avoiding a wider dissemination of angiogenic influence in the AC and the wound site.

The expression profiles of TGF-β isoforms in the rabbit aqueous humor is striking, with ratios heavily skewed towards TGFβ2 (4 TGF-β1: 800 TGF-β2: 1 TGF-β3). This is similar to that in humans, where TGF-β2 comprised the bulk of the isoforms [21, 22]. TGF-β2 was reported to be elevated in the aqueous humor of glaucoma patients [15]. Together with data correlating aqueous TGF-β2 with high IOP [23], and experimental evidence for the capacity of TGF-β2 to reduce outflow facility when perfused into cultured human anterior segments [24], the implication of aqueous TGF-β2 in the pathogenesis of the ocular hypertension in glaucoma is compelling. Our study revealed that neither ACP nor PI caused significant elevation in aqueous TGF-β2 levels.

On the other hand, aqueous TGF-β1 levels increased in response to ACP on POD 1, but this elevation was only made significant when PI was performed in conjunction with ACP. ACP may trigger TGF-β1 induction as this procedure is associated with increased inflammation. An experimental study on beagle eyes indicated that ACP caused significant increase in inflammatory markers in the canine aqueous humor within 4 hours [25]. This time frame may correspond with the increase in aqueous TGF-β1 shortly following ACP, and suggests that TGF-β1 may be a constituent of the cocktail of molecules induced as part of the inflammatory response to ACP. The extra push rendered by PI to cause significant increase of TGF-β1 above baseline suggests that concomitant iris injury adds to the rise of this cytokine. Interestingly, Tenon’s fibroblasts from pseudoexfoliation (PEX) syndrome/ glaucoma were demonstrated to have heightened sensitivity to TGF-β1 than TGF-β2 in relation to expression and synthesis of profibrotic markers [26]. It may therefore be tempting to speculate that, at least in PEX glaucoma, where aqueous TGF-β2 levels were normal [16] but TGF-β1 was elevated [27], there is the potential for further increase in aqueous TGF-β1 induced by ACP/ PI to raise the risk of unfavorable bleb development after surgery than other glaucomas.

TGF-β3 levels were also induced by PI in the rabbit aqueous humor. Again, the PI-associated significance suggests that the damaged iris is a source of the induced aqueous TGF-β3. However, it is unclear whether the iris is a depot of TGF-β3. Interrogation of the anterior segment by means of immunohistochemical evaluation failed to detect TGF-β3 [15]. Although measurable, mean TGF-β3 concentration detected in the rabbit aqueous humor was in the order of 10 pg/ml. Similarly, TGF-β3 in normal human aqueous was no higher than 10 pg/ml^21^, or below detectable level in at least one study [22], supporting the likelihood that TGF-β3 is present at physiologically low levels in the aqueous humor. Nevertheless, the induction of TGF-β3 in the aqueous may have interesting implications for wound healing, with direct impact on the damaged iris, and potential indirect effects on the postoperative conjunctiva after glaucoma surgery. A striking quality of the iris is its ability to resist abundant scarring after injury, as suggested by observations of limited scar formation in human iris subjected to surgical iridectomy [28]. While the mechanism for the ability of the iris to avoid a profound scar response remains unclear, the aqueous humor is speculated to be involved [29]. Our data suggest that increased aqueous TGF-β3 may be a candidate for proffering the anti-fibrotic response in the wounded iris. The basis for this conjecture is founded on cumulative experimental and clinical evidence that TGF-β3 has anti-fibrotic activities, particularly in reducing dermal scarring [30, 31]. It is therefore tempting to speculate that elevated levels of TGF-β3 induced by the wounded iris may be a protective strategy by preventing excessive scarring in response to certain traumas, such as surgical iridectomy. By extension, increased aqueous TGF-β3 induced by PI as part of glaucoma surgery may be expected to percolate into the conjunctival bleb and aid in the reduction of postoperative scarring. Yet, recombinant TGF-β3 was shown to stimulate the mouse conjunctival scarring response [12]. Contradictory experimental findings relating to the functions of TGF-β3 are not new, leading to the suggestion that TGF-β3 has complex roles [30, 32], which we predict to be tissue-specific and condition-dependent. It is possible that the distinct molecular environments of the wounded iris and the postoperative conjunctiva may provide opposite regulation of TGF-β3 activities such that divergent wound healing outcomes may ensue. Interestingly, like TGF-β1 [27], increased TGF-β3 was detected in the aqueous humor of PEX glaucoma eyes [21]. The further induction of not one, but these two specific TGF-β isoforms by ACP/ PI in the aqueous humor as part of glaucoma filtration surgery suggests the potential for even greater risk of postoperative scarring in the PEX glaucoma conjunctiva. Indeed, a recent comparative study of the long-term success rates of glaucoma surgeries with PI indicated lower success for PEX glaucoma than for primary open-angle glaucoma eyes [33]. Hence, the necessity of PI in glaucoma surgery, particularly for treatment of PEX glaucoma, deserve additional consideration with respect to the potential impact of TGF-β isoforms on postoperative scarring and long-term surgical success.

In summary, we have demonstrated that PI, as performed in a rabbit model, is associated with increased inflammation. While PI did not increase the amount of aqueous VEGF-A, it caused significant aqueous elevations of TGF-β1 and TGF-β3. The upregulation of TGF-β3 may have implication for reduced iris fibrosis and long-term PI patency, whereas increased aqueous TGF-β1 and TGF-β3 may promote scarring, especially in PEX glaucoma eyes with their apparent unique sensitivities to these isoforms. Although all PI-associated tissue and molecular responses were primarily acute and not sustained in this normal rabbit model, it may contribute to the increased wound healing response in glaucoma eyes undergoing filtration surgery.

## Data Availability

The datasets generated and analysed during the study are not publicly available but are available from the corresponding author on reasonable request.
